# Serum sodium to chloride ratio and mortality on continuous ambulatory peritoneal dialysis: a multicenter retrospective study

**DOI:** 10.1002/mco2.70041

**Published:** 2025-01-10

**Authors:** Jiayin You, Sijie Gu, Ning Su, Xiaoran Feng, Fenfen Peng, Qingdong Xu, Xiaojiang Zhan, Yueqiang Wen, Xiaoyang Wang, Na Tian, Xianfeng Wu, Niansong Wang

**Affiliations:** ^1^ Department of Nephrology Shanghai Jiao Tong University Affiliated Sixth People's Hospital Shanghai China; ^2^ Department of Nephrology The Sixth Affiliated Hospital of Sun Yat‐Sen University Guangzhou China; ^3^ Department of Nephrology Jiujiang NO.1 People's Hospital Jiujiang China; ^4^ Department of Nephrology Zhujiang Hospital Southern Medical University Zhujiang China; ^5^ Department of Nephrology Jiangmen Central Hospital Jiangmen China; ^6^ Department of Nephrology The First Affiliated Hospital of Nanchang University Nanchang China; ^7^ Department of Nephrology The Second Affiliated Hospital of Guangzhou Medical University Guangzhou China; ^8^ Department of Nephrology The First Affiliated Hospital of Zhengzhou University Zhengzhou China; ^9^ Department of Nephrology General Hospital of Ningxia Medical University Yinchuan China

**Keywords:** all‐cause mortality, cardiovascular mortality, peritoneal dialysis, sodium‐to‐chloride ratio

## Abstract

An imbalance in the serum sodium to chloride ratio (Na/Cl) was linked to higher mortality among heart failure patients. Nonetheless, the prognostic significance of Na/Cl in individuals undergoing peritoneal dialysis (PD) remains unexplored. This study seeks to explore the association between initial Na/Cl levels and mortality in PD patients. The study, conducted across multiple centers, included 3341 patients undergoing PD from January 1, 2005, to December 31, 2021. Patients were stratified into quartiles according to baseline Na/Cl and followed up for a median of 5.77 years. To explore the association between Na/Cl levels and mortality, we employed Cox proportional hazards models, competing risks models, and restricted cubic spline analysis. Of 3341 patients, 722 patients died, including 259 cardiovascular deaths. Following adjustments for comorbidities and multiple covariates, individuals in the highest Na/Cl quartile (>1.42) exhibited lower all‐cause mortality (hazard ratio [HR] 0.63, 95% confidence interval [CI] 0.47–0.86) and cardiovascular mortality (HR 0.38, 95% CI 0.22–0.67) compared with those in the lowest quartile (<1.33). A similar pattern was also found when Na/Cl was dealt with continuous variables. Initial levels of Na/Cl at the start of PD were negatively correlated with all‐cause mortality and cardiovascular mortality in PD patients.

## Graphical abstract

In this study, 3341 patients on peritoneal dialysis between 2005 and 2021 were enrolled and grouped into quartiles according to baseline Na/Cl. After following up for a median of 5.77 years, the results showed that the highest Na/Cl quartile patients were associated with lower all‐cause and cardiovascular mortality, compared with that of the lowest quartile patients.

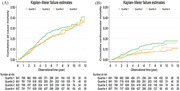

## 1 INTRODUCTION

End‐stage renal disease (ESRD) is a widespread condition that carries a significant risk of morbidity and mortality globally. In China, the estimated age‐adjusted incidence rate of dialysis was 122 per million general population annually.^1^ It is estimated that by 2030, the global utilization of renal replacement therapy will have increased by more than twofold, reaching 5.4 million individuals, with the most significant growth anticipated in Asia,^2^ which poses a great healthcare burden.

Electrolyte disturbances are one of the most common complications of ESRD.^3^, ^4^ Several studies have indicated that hyponatremia was associated with a higher risk of mortality in incident dialysis patients.^5^, ^6^, ^7^ When dialysis begins, the disturbance often improves as serum electrolyte levels are adjusted through the diffusion process of dialysis. Nevertheless, hyponatremia remains a mortality risk for patients both before and during dialysis.^4^, ^8^, ^9^, ^10^, ^11^, ^12^ Patients on peritoneal dialysis (PD) may also have a unique predisposition to dysnatremia, which can arise from a variety of causes. It can be reasonably deduced that the PD prescription for any given peritoneal solute transfer rate may serve as a significant predictor of the prevalence of hypo‐ or hypernatremia. It is also of significance to note that the absorption of glucose from dextrose‐based dialysate or icodextrin metabolites has the potential to result in a reduction in serum sodium levels.^13^ It remains unclear whether correcting hyponatremia improves patient outcomes.

Recent studies have also discovered that both low and high chloride levels were associated with higher all‐cause mortality and cardiovascular (CV) mortality in patients with PD.^14^, ^15^ Despite these studies indicating that serum chloride levels remained an independent mortality risk factor even after accounting for serum sodium, potassium, and other covariates, dyschloremia frequently occurs alongside dysnatremia, and the interaction between these ions in regulating electrolytes and acid‐base balance is intricate. Additionally, although some studies have shown that hyponatremia was linked to clinical outcomes, this association often attenuated or disappeared after adjustment with covariates including chloride levels.^16^, ^17^, ^18^, ^19^, ^20^

Given the strong correlation between serum sodium and chloride, it is challenging to entirely disregard one ion and simply consider the influence of another ion. Therefore, it is suggested that serum sodium and chloride should be evaluated together in patients with chronic kidney disease (CKD). Hence, a new index incorporating both serum sodium and chloride, namely the sodium‐to‐chloride ratio (Na/Cl) has gained increasing attention in recent studies. Studies indicated that both higher and lower baseline Na/Cl were associated with adverse outcomes and higher mortality rates in patients with heart failure.^21^, ^22^ Yet, no study has been conducted so far to assess the association of Na/Cl and PD patients. Therefore, the objective of this study is to evaluate the association between Na/Cl and mortality in patients on continuous ambulatory peritoneal dialysis (CAPD).

## 2 RESULTS

### 2.1 Baseline characteristics

We excluded 47 patients <18 years of age, 786 patients with pre‐existing CV diseases, 248 patients taking diuretics, 503 patients missing baseline serum sodium and chloride values, 88 patients with left‐censored data (missing time of death), 115 patients with less than 3‐month follow up. Therefore, 3341 patients were finally enrolled in the analysis (Figure S1). To demonstrate that the missing data were randomly distributed, the baseline characteristics of the 503 patients lacking sodium and chloride levels were compared with those of the 3341 patients included in the study (see Table S1). The results demonstrated that there was no statistically significant difference between the nonmissing Na/Cl and missing Na/Cl cohorts with regard to the majority of variables. This finding indicates that the missing data were randomly distributed.

Of the 3341 participants, there were 1886 patients with complete data on covariates, and baseline demographic and characteristic data of patients stratified by quartiles of Na/Cl and missing variables were shown in Table 1. Overall, the mean age was 48.95 ± 14.38 years, 55.9% were male, and 18.1% and 71.7% were complicated with diabetes and hypertension. The median Na/Cl was 1.38 (interquartile range [IQR] 1.33–1.42). There were no significant differences in age, gender, and the prevalence of hypertension and diabetes.

**TABLE 1:** Baseline characteristics of study population by Na/Cl ratio.

Variable	Total	Q1 (<1.33)	Q2 (1.34–1.37)	Q3 (1.38–1.42)	Q4 (>1.42)	Missing data, *n*	*p*‐value
*N*	3341	841	841	822	837	–	
Age, year	48.95 ± 14.38	50.8 ± 14.25	49.81 ± 14.08	50.08 ± 14.54	49.10 ± 14.60	0	0.699
Men, *n* (%)	1867 (55.9)	468 (55.7)	478 (56.8)	475 (57.8)	446 (53.4)	1	0.294
BMI, kg/m^2^	22.46 ± 6.75	22.24 ± 3.45	22.72 ± 7.01	22.56 ± 8.20	22.33 ± 7.26	215	<0.001
DM, *n* (%)	560 (18.1)	139 (16.9)	137 (17.5)	151 (20.5)	133 (17.8)	251	0.260
Hypertension, *n* (%)	2063 (71.7)	556 (70.6)	528 (71.7)	506 (75.2)	473 (69.5)	463	0.101
Albumin, g/L	35.23 ± 8.70	35.42 ± 5.86	35.14 ± 5.31	35.22 ± 5.32	35.12 ± 14.55	48	<0.001
eGFR, mL/min/1.73 m2	5.37 (3.97–7.28)	5.99 (4.64, 7.92)	5.55 (4.14, 7.78)	5.27 (3.94, 7.10)	4.56 (3.40, 6.16)	1	<0.001
Cholesterol, mmol/L	4.38 (3.59, 5.22)	4.11 (3.33, 4.90)	4.45 (3.69, 5.28)	4.50 (3.72, 5.32)	4.49 (3.76, 5.32)	357	<0.001
Calcium, mmol/L	2.12 (1.93, 2.28)	2.02 (1.82, 2.18)	2.13 (1.96, 2.29)	2.16 (2.00, 2.31)	2.16 (1.98, 2.33)	207	<0.001
Potassium, mmol/L	4.11 (3.60, 4.70)	4.50 (4.00, 5.10)	4.20 (3.70, 4.69)	3.98 (3.50, 4.51)	3.86 (3.40, 4.40)	0	<0.001
Sodium	140.3 (138.0, 142.7)	140.0 (138.0, 142.0)	140.4 (138, 142.3)	141.0 (139.0, 143.0)	140.3 (137.5, 143.0)	0	<0.001
Chloride	102.0 (98.4, 105.3)	108.0 (106.0, 111.0)	103.0 (101.8, 105.0)	100.7 (99.0, 102.0)	96.0 (94.0, 98.4)	0	<0.001
Na/Cl	1.38 (1.34, 1.42)	1.30 (1.27, 1.32)	1.36 (1.35, 1.37)	1.40 (1.39, 1.41)	1.45 (1.44, 1.48)	0	<0.001
LDL	2.53 (1.97, 3.20)	2.31 (1.80, 2.91)	2.56 (2.02, 3.25)	2.63 (2.08, 3.31)	2.64 (2.10, 3.31)	544	<0.001
HDL	1.10 (0.90, 1.40)	1.05 (0.87, 1.39)	1.10 (0.91, 1.39)	1.11 (0.91, 1.36)	1.13 (0.91, 1.45)	610	0.028
Total Kt/V	2.14 (1.73, 2.65)	2.10 (1.63, 2.68)	2.21 (1.79, 2.71)	2.20 (1.74, 2.66)	2.04 (1.73, 2.50)	911	0.001
Centers						0	<0.001
1	347 (10.4)	27 (3.21)	91 (10.82)	123 (14.96)	106 (12.66)		
2	883 (26.4)	481 (57.19)	220 (26.16)	127 (15.45)	55 (6.57)		
3	82 (2.5)	57 (6.78)	14 (1.66)	6 (0.73)	5 (0.6)		
4	681 (20.4)	68 (8.09)	162 (19.26)	210 (25.55)	241 (28.79)		
5	442 (13.2)	90 (10.7)	105 (12.49)	88 (10.71)	159 (19)		
6	108 (3.2)	5 (0.59)	14 (1.66)	21 (2.55)	68 (8.12)		
7	356 (10.7)	51 (6.06)	82 (9.75)	105 (12.77)	118 (14.1)		
8	442 (13.2)	62 (7.37)	153 (18.19)	142 (17.27)	85 (10.16)		

### 2.2 Na/Cl and mortality

#### 2.2.1 Patient outcomes

During 17,437.9 person‐years of follow‐up [median 5.77 (IQR 3.74, 7.89) years], 722 (21.6%) patients died, 360 (10.8%) patients transferred to hemodialysis, 170 (5.1%) patients underwent renal transplantation, and 23 (0.7%) patients transferred to other dialysis facilities. Of 722 deaths, 259 (35.9%) deaths were caused by CV diseases. Deaths occurred in 196 (46.7/1000 person‐years), 173 (39.4/1000 person‐years), 173 (39.1/1000 person‐years), and 180 (40.7/1000 person‐years) patients across the quartiles of the Na/Cl ratio from the lowest to the highest, respectively (Table 2).

**TABLE 2:** Incidence rate of death according to quartiles of Na/Cl ratio.

Outcomes	Overall	Q1 (<1.33)	Q2 (1.34–1.37)	Q3 (1.38–1.42)	Q4 (>1.42)
No. of patients	3341	841	841	822	837
Person‐years	17,437.9	4194.2	4390.0	4425.7	4428.0
Follow‐up years, median (IQR)	5.77 (3.74, 7.89)	5.83 (3.27, 8.23)	5.62 (3.68, 7.79)	5.79 (4.01, 7.96)	5.81 (4.01, 7.66)
All‐cause mortality					
Events	722	196	173	173	180
Events per 1000 person‐years	41.4	46.7	39.4	39.1	40.7
Cardiovascular mortality					
Events	259	90	70	50	49
Events per 1000 person‐years	14.9	21.5	15.9	11.3	11.1

#### 2.2.2 Kaplan–Meier curve analyses

**FIGURE 1:**
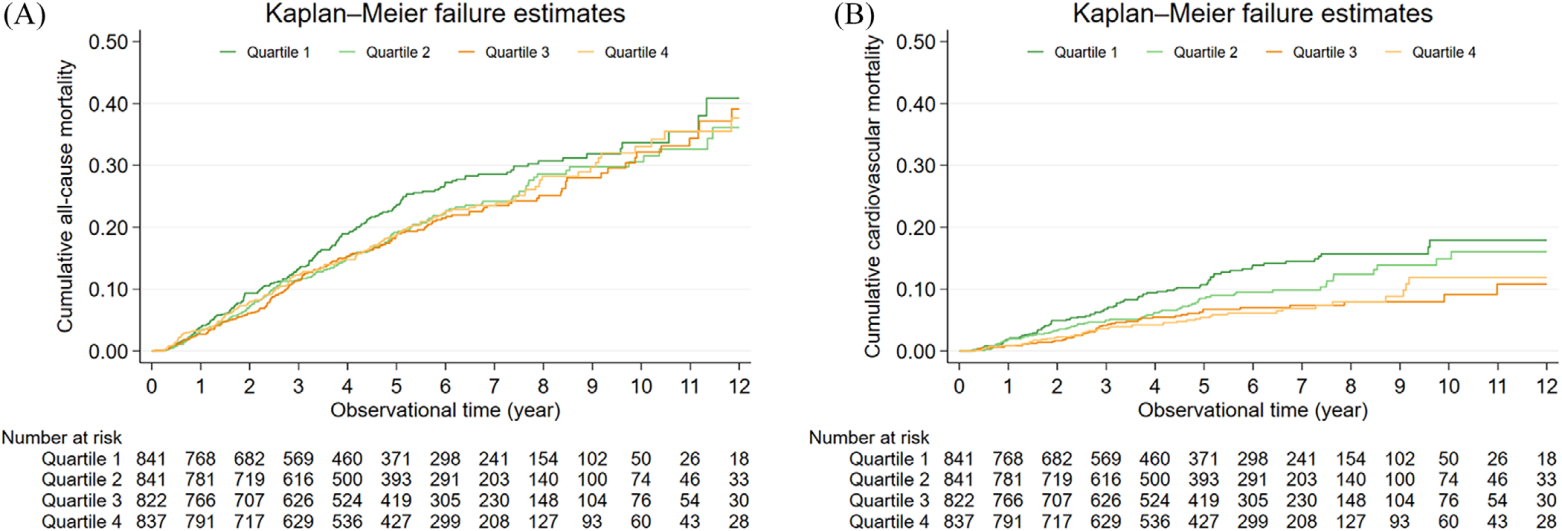
Cumulative incidence of all‐cause and cardiovascular mortality according to Na/Cl ratio quartile. *p*‐values of the log‐rank test for all‐cause mortality and cardiovascular mortality were 0.222 and <0.001, respectively.

To explore the association between Na/Cl and all‐cause mortality and CV mortality, analysis using Kaplan–Meier curves revealed a notable reduction in CV mortality in the highest quartile while all‐cause mortality did not differ significantly across the quartiles (Figure 1).

#### 2.2.3 Association between Na/Cl and all‐cause and CV mortality

To further assess the association between Na/Cl and mortality, cox proportional hazard model was utilized. Among the 1886 patients with complete covariate data, the unadjusted HRs (Model 1) for all‐cause mortality were 0.97 (95% CI, 0.73–1.27), 1.17 (95% CI, 0.91–1.52), and 1.00 (95% CI, 0.77–1.29) for the fourth, third, and second quartiles, respectively, in comparison with the first quartile. The results were similar after adjusting for demographics and comorbidities in Model 2. Nevertheless, following further adjustments with covariables in the ultimate model (Model 3), the HR for all‐cause mortality in the top quartile of Na/Cl was 0.63 (95% CI, 0.47–0.86) relative to the bottom quartile. The association between Na/Cl and CV mortality was more significant, in which the highest quartile of Na/Cl was associated with a 62% (95% CI, 0.22–0.67) lower risk of CV mortality after adjustments with demographics, comorbidities, and covariables in Model 3 compared with that of the lowest quartile (Table 3). In order to consolidate our results, we further did multiple imputations to impute missing data on covariates. Based on 3341 patients with imputed data, we found comparable outcomes for the relationships between Na/Cl and both all‐cause and CV mortality. The adjusted HRs for all‐cause and CV mortality were 0.69 (95% CI, 0.56–0.85) and 0.44 (95% CI, 0.31–0.65) for the highest quartile, respectively (Table S2).

**TABLE 3:** Association between Na/Cl ratio and mortality using Cox proportional hazard models based on complete data (*n* = 1886).

	Model 1	*p*‐value	Model 2	*p*‐value	Model 3	*p*‐value
All‐cause mortality						
Q1 (<1.33)	Reference	–	Reference	–	Reference	–
Q2 (1.34–1.37)	1.00 (0.77–1.29)	0.979	1.06 (0.82–1.37)	0.641	0.73 (0.56–0.96)	0.025
Q3 (1.38–1.42)	1.17 (0.91–1.52)	0.222	1.31 (1.01–1.69)	0.040	0.82 (0.62–1.08)	0.153
Q4 (>1.42)	0.97 (0.73–1.27)	0.807	1.10 (0.84–1.45)	0.497	0.63 (0.47–0.86)	0.003
*p* for trend^a^		0.829		0.210		0.008
Na/Cl ratio per 0.1 increase	0.92 (0.81–1.05)	0.205	1.01 (0.88–1.15)	0.914	0.71 (0.60–0.83)	<0.001
Cardiovascular mortality						
Q1 (<1.33)	Reference	–	Reference	–	Reference	–
Q2 (1.34–1.37)	0.91 (0.62–1.34)	0.638	0.96 (0.66–1.42)	0.851	0.76 (0.51–1.14)	0.188
Q3 (1.38–1.42)	0.86 (0.57–1.29)	0.468	0.96 (0.63–1.44)	0.833	0.71 (0.45–1.11)	0.131
Q4 (>1.42)	0.48 (0.29–0.80)	0.005	0.56 (0.34–0.93)	0.025	0.38 (0.22–0.67)	0.001
*p* for trend^a^		0.009		0.056		0.001
Na/Cl ratio per 0.1 increase	0.72 (0.58–0.89)	0.003	0.78 (0.62–0.97)	0.027	0.60 (0.46–0.78)	<0.001

*Note*: Model 1: unadjusted crude HR. Model 2: adjusted for age, sex, BMI, DM, and hypertension. Model 3: model 2 plus albumin, eGFR, cholesterol, calcium, potassium, HDL, LDL, Total Kt/V, and centers.

Abbreviations: BMI, body mass index; CI, confidence interval; DM, diabetes mellitus; eGFR, estimated glomerular filtration rate; HDL, high‐density cholesterol; HR, hazards ratio; LDL, low‐density cholesterol; Q, quartile.

^a^ Test for trend based on the variable containing a median value for each quartile.

Additionally, subdistribution hazard models were performed to validate the primary analysis involving 1886 patients with complete data. The HRs for all‐cause mortality and CV mortality in the highest Na/Cl quartile were 0.73 (95% CI, 0.54–1.00) and 0.44 (95% CI, 0.25–0.77), respectively, compared with the lowest quartile, after accounting for demographics, comorbidities, and covariables in Model 3 (Table 4). Analyzing data from 3341 patients with imputed data, we found comparable outcomes concerning the associations between Na/Cl and all‐cause and CV mortality. The adjusted HRs for all‐cause and CV mortality were 0.75 (95% CI, 0.60–0.92) and 0.49 (95% CI, 0.33–0.71) for the fourth quartile, respectively (Table S3). Furthermore, since non‐CV mortality was treated as a competing risk for CV mortality, competing risk models were employed to evaluate the association between Na/Cl and CV mortality. As shown in Table 5 and Table S4, the adjusted HRs for risk of CV mortality in the highest quartile were 0.43 (95% CI, 0.25–0.73) and 0.48 (95% CI, 0.33–0.69), based on complete and imputed data, respectively, which further corroborated our previous analysis.

**TABLE 4:** Association between Na/Cl ratio and mortality using sub‐distribution hazard models based on complete data (*n* = 1886).

	Model 1	*p*‐value	Model 2	*P*‐value	Model 3	*p*‐value
All‐cause mortality						
Q1 (<1.33)	Reference	–	Reference	–	Reference	–
Q2 (1.34–1.37)	1.03 (0.80–1.33)	0.817	1.11 (0.86–1.43)	0.426	0.83 (0.63–1.10)	0.190
Q3 (1.38–1.42)	1.21 (0.93–1.56)	0.153	1.35 (1.05–1.75)	0.021	0.95 (0.72–1.26)	0.718
Q4 (>1.42)	0.98 (0.75–1.29)	0.888	1.13 (0.86–1.49)	0.381	0.73 (0.54–1.00)	0.052
*p* for trend^a^		0.731		0.140		0.112
Na/Cl ratio per 0.1 increase	0.93 (0.82–1.05)	0.237	1.02 (0.9–1.16)	0.745	0.80 (0.68–0.93)	0.005
Cardiovascular mortality						
Q1 (<1.33)	Reference	–	Reference	–	Reference	–
Q2 (1.34–1.37)	0.94 (0.64–1.38)	0.761	1 (0.68–1.47)	0.982	0.86 (0.57–1.31)	0.485
Q3 (1.38–1.42)	0.88 (0.59–1.33)	0.552	0.99 (0.65–1.49)	0.945	0.82 (0.52–1.29)	0.392
Q4 (>1.42)	0.49 (0.29–0.81)	0.005	0.57 (0.34–0.94)	0.028	0.44 (0.25–0.77)	0.004
*p* for trend^a^		0.011		0.068		0.007
Na/Cl ratio per 0.1 increase	0.73 (0.59–0.9)	0.003	0.79 (0.63–0.98)	0.035	0.67 (0.51–0.87)	0.003

*Note*: Model 1: unadjusted crude HR. Model 2: adjusted for age, sex, BMI, DM, and hypertension. Model 3: model 2 plus albumin, eGFR, cholesterol, calcium, potassium, HDL, LDL, total Kt/V, and centers.

Abbreviations: CI, confidence interval; BMI, body mass index; DM, diabetes mellitus; eGFR, estimated glomerular filtration rate; HDL, high‐density cholesterol; HR, hazards ratio; LDL, low‐density cholesterol; Q, quartile.

^a^ Test for trend based on the variable containing a median value for each quartile.

**TABLE 5:** Association between Na/Cl ratio and cardiovascular mortality using competing risk models based on complete data (*n* = 1886).

	Model 1	*p*‐value	Model 2	*p*‐value	Model 3	*p*‐value
Q1 (<1.33)	Reference	–	Reference	–	Reference	–
Q2 (1.34–1.37)	0.91 (0.62–1.33)	0.621	0.94 (0.64–1.37)	0.737	0.82 (0.54–1.23)	0.329
Q3 (1.38–1.42)	0.83 (0.55–1.25)	0.366	0.89 (0.59–1.34)	0.585	0.76 (0.49–1.18)	0.225
Q4 (>1.42)	0.47 (0.28–0.78)	0.003	0.52 (0.31–0.87)	0.012	0.43 (0.25–0.73)	0.002
*p* for trend^a^		0.004		0.018		0.002
Na/Cl ratio per 0.1 increase	0.71 (0.59–0.87)	0.001	0.75 (0.61–0.93)	0.007	0.66 (0.52–0.84)	0.001

*Note*: Model 1: unadjusted crude HR. Model 2: adjusted for age, sex, BMI, DM, and hypertension. Model 3: model 2 plus albumin, eGFR, cholesterol, calcium, potassium, HDL, LDL, total Kt/V, and centers.

Abbreviations: CI, confidence interval; BMI, body mass index; DM, diabetes mellitus; eGFR, estimated glomerular filtration rate; HDL, high‐density cholesterol; HR, hazards ratio; LDL, low‐density cholesterol; Q, quartile.

^a^ Test for trend based on the variable containing a median value for each quartile.

Since the time of death was unavailable for 88 patients, who were considered as left‐censored data, we additionally employed parametric models for interval‐censored survival‐time data (stintreg) to validate our findings. Left‐censored data with complete details on covariables were added to the original 1886 patients, hence a total of 1902 patients were included in the analysis. According to Table S5, the adjusted HRs for all‐cause and CV mortality in the highest quartile were 0.73 (95% CI, 0.53–0.99) and 0.46 (95% CI, 0.26–0.80), respectively. After multiple imputations, 88 left‐censored data were added to 3341 patients. Comparable trends in mortality were observed, with the adjusted HRs for all‐cause and CV mortality in the highest quartile being 0.74 (95% CI, 0.60–0.91) and 0.52 (95% CI, 0.36–0.76), respectively (Table S6).

**FIGURE 2:**
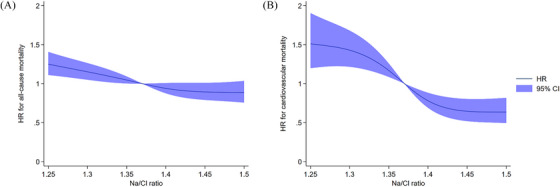
Association between Na/Cl ratio as a continuous variable and the risk of mortality using RCS models (based on imputed data).

Ultimately, based on imputed data, when Na/Cl was analyzed as a continuous variable in the restricted cubic spline models, we found graded and inverse associations between Na/Cl and all‐cause and CV mortality, in which the trend for CV mortality is more prominent (Figure 2).

### 2.3 Subgroup analysis

**FIGURE 3:**
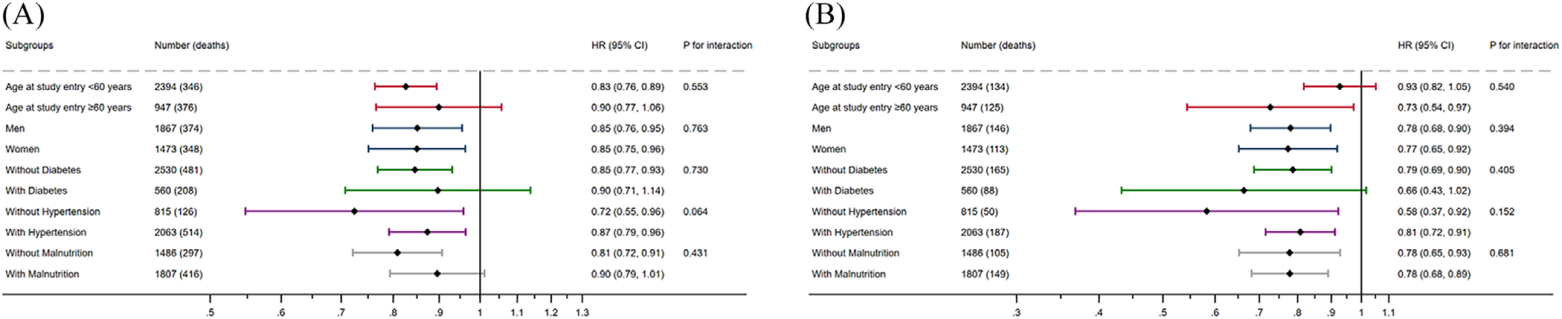
Subgroup association of baseline continuous Na/Cl ratio with all‐cause and cardiovascular mortality.

Subgroup analysis was subsequently performed based on categories of age, sex, comorbidities, and albumin levels. The associations between Na/Cl and all‐cause mortality and CV mortality remained consistent across different subgroups, in which the trends for CV mortality across different subgroups were more pronounced (Figure 3).

### 2.4 Sensitivity analysis

A sensitivity analysis was conducted on patients who did not experience mortality during the initial 2‐year follow‐up period or the follow‐up period exceeding 24 months. It is noteworthy that the risks of both all‐cause and CV mortality were lower in the second, third, and fourth quartiles compared with the first quartile (see Tables S7 and S8).

## 3 DISCUSSION

The results of this multicenter retrospective cohort study comprising 3341 PD patients demonstrated a significant inverse correlation between baseline Na/Cl levels and all‐cause mortality and CV mortality at 12 years. After adjustments with demographics and covariables, compared with patients with Na/Cl < 1.33, those with Na/Cl > 1.42 were associated with 26% and 52% lower risk of all‐cause mortality and CV mortality, respectively. Subgroup analysis showed that age, sex, and comorbidities such as hypertension, diabetes, and malnutrition had no interaction with Na/Cl. The findings were robust due to the consistency of the results across different hazard models and sensitivity analyses. Our previous study which enrolled 2376 incident PD patients showed that higher baseline chloride levels and lower sodium levels at the initial of PD were associated with increased mortality.^14^ Furthermore, a substantial body of evidence indicates that lower serum sodium levels are associated with an elevated risk of mortality.^4^, ^6^, ^7^, ^11^ Therefore, our present study showing that lower baseline Na/Cl was associated with increased mortality was reliable and was the first study investigating the associations between Na/Cl and mortality in PD patients. Furthermore, measurements of serum sodium and chloride levels are routine clinical practice, which is economical and accessible. Close monitoring of this value and modifying it through diet can help us better manage these patients.

The relationship between serum sodium levels and mortality in patients with CKD and those undergoing dialysis has been the subject of numerous previous studies, with a high degree of consistency in the results. These studies have consistently demonstrated that lower serum sodium levels are associated with an increased risk of mortality.^4^, ^5^, ^6^, ^7^, ^8^, ^9^, ^10^, ^11^, ^12^ A single retrospective study comprising 16,483 participants identified an association between a low level of serum sodium and an elevated risk of CV mortality in healthy participants.^23^ A lower serum chloride level did not correlate with an increased risk of CV mortality. Additionally, a retrospective study of 32,666 participants demonstrated a U‐shaped relationship between serum sodium levels and mortality. Optimal serum sodium threshold values associated with the lowest mortality were found to range between 139 and 144 mmol/L.^24^ In the present study, the median serum sodium level in the total patient cohort was 140.3 (138.0, 142.7) mmol/L. Therefore, the serum sodium levels observed in our study were predominantly within the normal range. Studies investigating the association between serum chloride and mortality yielded inconsistent results. Previous studies have shown that low serum chloride was associated with an increased risk of mortality in heart failure,^16^, ^18^, ^22^ hypertension,^25^ pulmonary artery hypertension,^26^ and critical illness.^27^ In regard to patients with CAPD, a retrospective study comprising 1656 incident patients revealed that lower serum chloride levels were predictive of an elevated risk of all‐cause and CV mortality.^15^ However, our previous multicenter retrospective study encompassing 2376 incident PD patients revealed that higher serum chloride at the initial of PD was associated with a higher risk of all‐cause and CV mortality and that serum chloride was positively correlated with mortality in a linear pattern.^14^ From a physiological perspective, sodium and chloride are the most prevalent cations and anions within the human body, and chloride is considered a counter‐anion of sodium. It has been demonstrated that there is a notable correlation between serum sodium and chloride levels.^22^, ^28^, ^29^ In one study that enrolled 7195 patients with myocardial infarction complicated by reduced ejection fraction and heart failure, a notable interaction between sodium and chloride was observed. In this context, a serum chloride level of less than 97 mmol/L was associated with mortality, but not with hospitalization for heart failure, when sodium levels were lower. It can be hypothesized that patients with more severe congestion would more frequently present with dilutional hyponatremia, which, as previously stated, is associated with a poorer prognosis. Furthermore, these patients are more likely to be administered higher doses of diuretics (in an attempt to relieve congestion), which may induce (or exacerbate) hypochloremia. This proposed mechanism may offer an explanation for the worse prognosis observed in patients with both lower sodium and low chloride levels. Therefore, these data support an integrated consideration of sodium and chloride interplay.

Recently, a new marker combining the information of two ions, namely Na/Cl, has gained increasing attention. In one study that enrolled 1021 consecutive chronic heart failure (CHF) patients, admission chloride levels were inversely correlated with all‐cause mortality. However, when adjusting with sodium concentrations, the independent prognostic role of chloride disappeared, suggesting that chloride and sodium were closely related. The authors further discovered that reduced serum chloride levels may elevate the risk ratio of patients with CHF and hyponatremia. Additionally, higher Na/Cl was associated with a higher risk of mortality in CHF patients.^22^ Another study which included 1819 elderly acute heart failure patients found that both high and low Na/Cl ratios on admission were associated with a higher risk of all‐cause mortality. The authors suggested that lower Na/Cl represented a state of volume overload and congestion while higher Na/Cl represented a state of ion depletion, which could all influence the prognosis of patients.^21^ However, patients with CKD were more susceptible to fluid and electrolyte disturbances with metabolic acidosis compared to those with normal kidney function,^30^ while metabolic alkalosis was the most prevalent acid‐base disturbance in patients with heart failure.^31^ Therefore, inconsistent results may exist in patients with CKD when investigating the prognostic role of Na/Cl. Our previous work has suggested that both higher baseline chloride levels and lower sodium levels were associated with an increased risk of mortality in CAPD patients.^14^ The present study, which is the first to investigate the association of Na/Cl and mortality in CAPD patients, further corroborated the findings of previous studies that the lowest quartile of Na/Cl is associated with an increased risk of all‐cause and CV mortality. Furthermore, this association was more prominent after adjustments with other covariates. Based on previous studies mentioned above, a low Na/Cl ratio (<1.33) may represent a state of congestion and dilutional hyponatremia, both of which were closely related to increased risk of mortality. The subgroup analysis also highlights the consistency in the association between baseline Na/Cl and the risk of all‐cause and CV mortality across different subgroups. Despite the observed variation in the strength of the association between Na/Cl and the risk of all‐cause mortality across different age groups, diabetic patients, and malnourished individuals, the association between Na/Cl and CV mortality remained highly consistent. This suggests that higher sodium and chloride levels are associated with lower CV mortality across diverse patient subgroups. Although our present study showed that the mortality risk in the second and third Na/Cl quartiles also decreased compared with that of the first quartile after adjustments. We further took Na/Cl as a continuous variable for restricted cubic spline analysis. Restricted cubic analysis more accurately showed that, with increasing Na/Cl, the risk of all‐cause and CV mortality decreased, with the trend for CV mortality being more pronounced. In this context, our results were solid and appealing.

As part of routine clinical practice, measurement of serum electrolytes may assist nephrologists and other healthcare providers in identifying populations at risk of complications in patients undergoing CAPD. The findings of our study indicated that a lower Na/Cl ratio (less than 1.33) was associated with an elevated risk of mortality. This may be indicative of a state of congestion. Further studies are warranted to investigate whether correcting low Na/Cl levels either by restricting fluid intake or by aggressive diuresis will improve patient survival.

It should be noted that our study is subject to a number of limitations. First, due to the retrospective and observational nature of our study, it is not possible to discount the possibility of residual or unmeasured confounding, and therefore we cannot conclude that there is a direct causal relationship between Na/Cl and mortality. Second, due to single measurements of baseline sodium and chloride, bias from regression toward the mean may exist and whether changes of Na/Cl will influence the prognosis of patients are unclear and need further study. Third, it should be noted that our study was conducted exclusively with Chinese patients, and thus the findings may not apply to other populations. Fourth, the advancement of medication and nursing measures may also affect the analysis of mortality. Furthermore, complete exclusion of patients taking diuretics would reduce the generalizability of the conclusions. Further studies, including large‐scale prospective trials, are required to confirm our findings. Additionally, mechanistic studies are necessary to provide a comprehensive explanation of the results observed in our study.

## 4 CONCLUSION

In conclusion, the baseline sodium‐to‐chloride ratio at the commencement of CAPD was inversely correlated with all‐cause mortality and CV mortality in patients undergoing PD. Our findings may have translational significance in screening potentially high‐risk populations in patients undergoing CAPD. Further studies are needed to investigate whether correcting this electrolyte ratio will improve patient survival.

## 5 MATERIALS AND METHODS

### 5.1 Study design and population

The manuscript was prepared in accordance with the STROBE guidelines. This study was a retrospective cohort analysis of 5128 consecutive incident patients who began CAPD as their initial renal replacement therapy across eight tertiary hospitals in China, spanning from January 1, 2005, to December 31, 2021. Based on our previous study about serum chloride and mortality in PD patients,^14^ we applied identical exclusion criteria, excluding individuals aged <18 years, those with <3 months of follow‐up, pre‐existing CV disease, diuretic use, missing serum sodium and chloride, missing other covariables, and lost to follow‐up. The study protocol was conducted in accordance with the declaration of Helsinki and received approval from all relevant Clinical Research Ethics Committees. The data were de‐identified, and the requirement for informed consent was dismissed.

### 5.2 Data collection and measurements

Patients were recommended to begin dialysis at the discretion of professional nephrologists at each participating center. At the start, information on demographics, comorbid conditions, and lab results were collected for every patient, including age, gender, body mass index, diabetes mellitus, hypertension, albumin levels, estimated glomerular filtration rate, total cholesterol, high‐density lipoprotein, low‐density lipoprotein, sodium, chloride, potassium, calcium and hypersensitive C‐reactive protein (hs‐CRP). All laboratory tests were conducted in the laboratory of each participating center. All CAPD patients utilized conventional dialysis solutions (Dianeal 1.5%, 2.5%, or 4.25% dextrose; Baxter Healthcare, Guangzhou, China), along with Y sets, and twin bag systems.

### 5.3 Follow‐up and outcomes

It was mandatory for patients to be readmitted to each center on a quarterly basis for an overall medical assessment. Furthermore, a monthly face‐to‐face or telephone interview was conducted by a professional nurse in order to evaluate the patient's general condition and adherence to their medication regimen. Each patient was followed up until the date of death, transition to hemodialysis, undergoing renal transplantation, loss of follow‐up, transfer to another facility, or the end of the study period (December 31, 2021). Patients who were lost to follow‐up were considered to have reached the end of the study period at the date of their final evaluation.

The outcomes were all‐cause mortality and CV mortality, respectively. The cause of death was determined based on the medical records of admission. When the patient passed away outside the hospital, the cause of death was identified by interviewing family members to evaluate the death's manifestations, combined with prior medical records. CV death encompassed fatalities associated with acute coronary syndrome, heart failure, ischemic or hemorrhagic stroke, life‐threatening arrhythmia, and sudden cardiac death, as defined by the literature.^32^

### 5.4 Statistical analysis

For continuous variables that were roughly normally distributed, means and standard deviations were presented, while medians and IQR were shown for those with skewed distributions. Categorical variables were presented as the number of patients and percentage. To address the missing data on covariates under the assumption of a missing‐at‐random mechanism, we performed multiple imputations and utilized chained equations to generate 20 imputed data files. Continuous variables were imputed using linear regression models, while binary variables were imputed using logistic regression.

To investigate the association between Na/Cl ratio and mortality, cause‐specific hazard models (stcox) were employed. Subsequently, subdistribution hazard (streg, distribution) models were performed to validate the association identified in the primary analysis. The occurrence of noncardiovascular mortality was regarded as a competing risk for CV mortality (stcrreg function). These models were performed based on complete data and imputed data separately. Restricted cubic splines (xblc) were employed to examine the relationship between the Na/Cl ratio as a continuous variable and the risk of mortality. Furthermore, we incorporated 88 left‐censored data and employed parametric models for interval‐censored survival‐time data (stintreg) to verify the association between Na/Cl ratio and mortality.

Subgroup analyses were performed based on confounders such as age (<60 or ≥60 years old), gender (male or female), diabetes (present or absent), hypertension (present or absent), and malnutrition (malnourished with albumin <36.0 g/L or well‐nourished albumin ≥36.0 g/L). The statistical analyses were conducted using Stata 17.0 statistical software (StataCorp), and a *p*‐value of less than 0.05 was considered statistically significant.

## AUTHOR CONTRIBUTIONS

Jiayin You, Sijie Gu, Xianfeng Wu, and Niansong Wang were instrumental in the conceptualization of the research, the statistical analysis, the drafting, the revision, and the approval of the manuscript. Ning Su, Xiaoran Feng, Fenfen Peng, Qingdong Xu, Xiaojiang Zhan, Yueqiang Wen, Xiaoyang Wang, and Na Tian contributed to the interpretation of the results, revision of the manuscript, and approval of the final version. Jiayin You, Sijie Gu, and Xianfeng Wu accessed and verified the data. Niansong Wang is the guarantor of this study and accepts responsibility for the integrity of the data and the accuracy of the analysis. All authors have read and approved the final manuscript.

## CONFLICT OF INTEREST STATEMENT

The authors declare no conflict of interest.

## ETHICS STATEMENT

The study protocol was conducted in accordance with the Declaration of Helsinki and received approval from each Clinical Research Ethics Committee (approval number: hg‐ks‐11). The data were de‐identified, and the requirement for informed consent was waived.

## Supporting information



Supporting Information

## Data Availability

The entirety of the data generated or analyzed during the course of this study is included in this article and its supplementary material files. Should further inquiries arise, they should be directed to the corresponding author.
