# Supplementary material for: Serum sodium to chloride ratio and mortality on continuous ambulatory peritoneal dialysis: a multicenter retrospective study
Source: MedComm (2020). 2025 Jan 10;6(1):e70041. doi: 10.1002/mco2.70041 (PMC11718323; doi:10.1002/mco2.70041)
Supplement: Supplementary file 1 — Supporting Information [file MCO2-6-e70041-s001.docx]

**Serum sodium to chloride ratio and mortality on continuous ambulatory peritoneal dialysis: a multi-center retrospective study**

**Running title: Na/Cl predicts prognosis in PD patients**

Jiayin You^1#^, Sijie Gu^1#^, Ning Su^2^, Xiaoran Feng^3^, Fenfen Peng^4^, Qingdong Xu^5^, Xiaojiang Zhan^6^, Yueqiang Wen^7^, Xiaoyang Wang^8^, Na Tian^9^, Xianfeng Wu^1*^, Niansong Wang^1*^

|  |
| --- |

^1^ Department of Nephrology, Shanghai Jiao Tong University Affiliated Sixth People’s Hospital, Shanghai, China

^2^ Department of Nephrology, The Sixth Affiliated Hospital of Sun Yat-Sen University, Guangzhou, China

^3^ Department of Nephrology, Jiujiang NO.1 People’s Hospital, Jiujiang, China

^4^ Department of Nephrology, Zhujiang Hospital, Southern Medical University, Zhujiang, China

^5^ Department of Nephrology, Jiangmen Central Hospital, Jiangmen, China

^6^ Department of Nephrology, The First Affiliated Hospital of Nanchang University, Nanchang, China

^7^ Department of Nephrology, The Second Affiliated Hospital of Guangzhou Medical University, Guangzhou, China

^8^ Department of Nephrology, The First Affiliated Hospital of Zhengzhou University, Zhengzhou, China

^9^ Department of Nephrology, General Hospital of Ningxia Medical University, Yinchuan, China

**Correspondence**

Xianfeng Wu and Niansong Wang, Shanghai Jiao Tong University Affiliated Sixth People’s Hospital, Shanghai 200233, China.

E-mail: xianfengwu2@163.com (X. Wu); wangniansong2012@163.com (N.Wang)

Jiayin You and Sijie Gu contributed equally to this work.

**Supplementary information**

**Figure S1**

**Table S1-S8**

**Supplementary information**

**Figure S1.** Flow-chart of eligible and ineligible patients. The numbers of potential and eligible patients were shown on the left side, and the reasons for in ineligibility and the numbers of ineligible patients were shown on the right side. CAPD, continuous ambulatory peritoneal dialysis; CVD, cardiovascular disease.


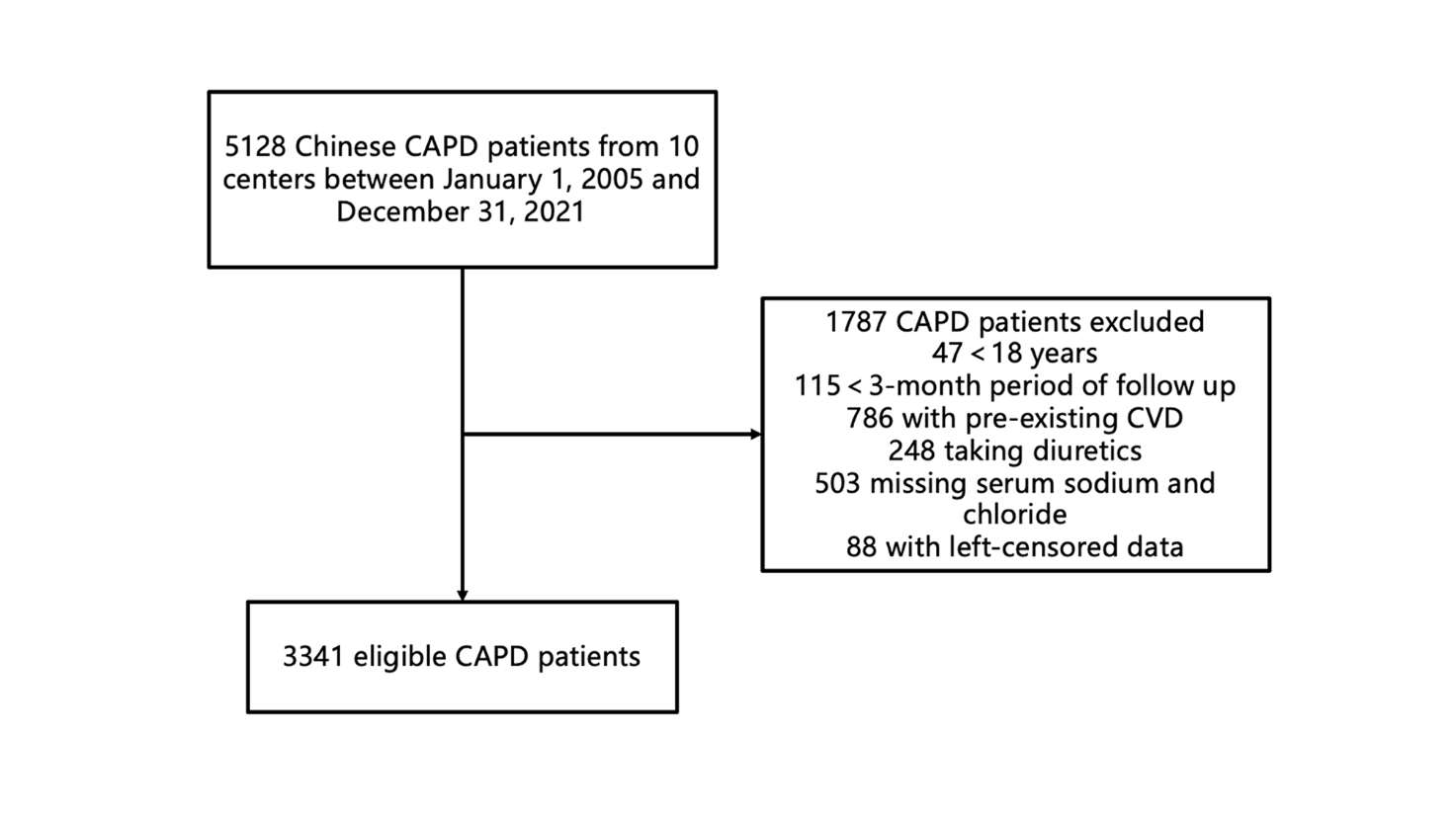


1. Baseline characteristics were compared between 503 patients missing sodium and chloride levels and 3341 patients who were enrolled in the study and associations between Na/Cl ratio and mortality based on imputed data..

Table S1. Baseline characteristics comparing 3341 patients with 503 patients missing sodium and chloride levels.

| Variable | Non-missing Na/Cl ratio | Missing Na/Cl ratio | *P*-value |
| --- | --- | --- | --- |
| N | 3341 | 503 |  |
| Age, year | 49.95±14.38 | 53.56±15.97 | <0.001 |
| Men, n (%) | 1867 (55.9) | 291 (58) | 0.383 |
| BMI, kg/m^2^ | 22.46±6.75 | 22.73±3.45 | 0.440 |
| DM, n (%) | 560 (18.1) | 89 (22.1) | 0.051 |
| Hypertension, n (%) | 2063 (71.7) | 124 (81.1) | 0.012 |
| Albumin, g/L | 35.23±8.7 | 32.46±6.39 | <0.001 |
| eGFR, mL/min/1.73m^2^ | 5.37 (3.97-7.28) | 4.88 (3.73-6.84) | 0.057 |
| Cholesterol, mmol/L | 4.38 (3.59-5.22) | 4.54 (3.82-5.53) | 0.107 |
| Calcium, mmol/L | 2.12 (1.93-2.28) | 2.19 (2.08-2.31) | <0.001 |
| Potassium, mmol/L | 4.11 (3.6-4.7) | 4.09 (3.62-4.74) | 0.918 |
| LDL | 2.53 (1.97-3.2) | 2.24 (1.67-2.95) | 0.223 |
| HDL | 1.1 (0.9-1.4) | 1.33 (1-1.67) | 0.101 |
| Total Kt/V | 2.14 (1.73- 2.65) | 1.65 (1.43- 2.24) | 0.004 |
| Centers |  |  | <0.001 |
| 1 | 347 (10.4) | 24 (4.8) |  |
| 2 | 883 (26.4) | 7 (1.4) |  |
| 3 | 82 (2.5) | 0 (0) |  |
| 4 | 681 (20.4) | 20 (4) |  |
| 5 | 442 (13.2) | 63 (12.5) |  |
| 6 | 108 (3.2) | 0 (0) |  |
| 7 | 356 (10.7) | 389 (77.3) |  |
| 8 | 442 (13.2) | 0 (0) |  |

| Table S2. Association between Na/Cl ratio and mortality using Cox proportional hazard models based on imputed data (n=3341). | | | | | | |
| --- | --- | --- | --- | --- | --- | --- |
|  | **Model 1** | ***P*-value** | **Model 2** | ***P*-value** | **Model 3** | ***P*-value** |
| All-cause mortality |  |  |  |  |  |  |
| Q1 (<1.33) | Reference | - | Reference | - | Reference | - |
| Q2 (1.34-1.37) | 0.83 (0.68-1.02) | 0.083 | 0.83 (0.67-1.02) | 0.071 | 0.71 (0.57-0.87) | 0.001 |
| Q3 (1.38-1.42) | 0.83 (0.68-1.02) | 0.075 | 0.81 (0.66-1.00) | 0.047 | 0.68 (0.55-0.85) | <0.001 |
| Q4 (>1.42) | 0.86 (0.70-1.05) | 0.138 | 0.89 (0.73-1.09) | 0.275 | 0.69 (0.56-0.85) | 0.001 |
| *P* for trend^*^ |  | 0.130 |  | 0.084 |  | 0.001 |
| Na/Cl ratio per 0.1 increase | 0.91 (0.84-0.98) | 0.013 | 0.92 (0.84-1.00) | 0.055 | 0.85 (0.80-0.92) | <0.001 |
| Cardiovascular mortality |  |  |  |  |  |  |
| Q1 (<1.33) | Reference | - | Reference | - | Reference | - |
| Q2 (1.34-1.37) | 0.74 (0.54-1.01) | 0.059 | 0.73 (0.54-1.00) | 0.051 | 0.66 (0.48-0.92) | 0.013 |
| Q3 (1.38-1.42) | 0.52 (0.37-0.74) | <0.001 | 0.50 (0.35-0.71) | <0.001 | 0.44 (0.31-0.64) | <0.001 |
| Q4 (>1.42) | 0.51 (0.36-0.73) | <0.001 | 0.53 (0.37-0.75) | <0.001 | 0.44 (0.31-0.65) | <0.001 |
| *P* for trend^*^ |  | <0.001 |  | <0.001 |  | <0.001 |
| Na/Cl ratio per 0.1 increase | 0.83 (0.75-0.91) | <0.001 | 0.81 (0.73-0.90) | <0.001 | 0.79 (0.72-0.87) | <0.001 |
| Model 1: unadjusted crude HR. Model 2: adjusted for age, sex, BMI, DM, and hypertension. Model 3: model 2 plus albumin, eGFR, cholesterol, calcium, potassium, HDL, LDL, Total Kt/V and centers. Q, quartile; BMI, body mass index; DM, diabetes mellitus; eGFR, estimated glomerular filtration rate; HDL, high-density cholesterol; LDL, low-density cholesterol; HR, hazards ratio; CI, confidence interval. ^*^, test for trend based on variable containing median value for each quartile. | | | | | | |

| Table S3. Association between Na/Cl ratio and mortality using sub-distribution hazard models based on imputed data (n=3341). | | | | | | |
| --- | --- | --- | --- | --- | --- | --- |
|  | **Model 1** | ***P*-value** | **Model 2** | ***P*-value** | **Model 3** | ***P*-value** |
| All-cause mortality |  |  |  |  |  |  |
| Q1 (<1.33) | Reference | - | Reference | - | Reference | - |
| Q2 (1.34-1.37) | 0.84 (0.69-1.03) | 0.098 | 0.83 (0.67-1.02) | 0.070 | 0.71 (0.58-0.88) | 0.002 |
| Q3 (1.38-1.42) | 0.83 (0.68-1.02) | 0.080 | 0.81 (0.66-1.00) | 0.046 | 0.70 (0.57-0.87) | 0.001 |
| Q4 (>1.42) | 0.87 (0.71-1.06) | 0.171 | 0.90 (0.74-1.11) | 0.327 | 0.75 (0.60-0.92) | 0.007 |
| *P* for trend^*^ |  | 0.156 |  | 0.998 |  | 0.007 |
| Na/Cl ratio per 0.1 increase | 0.91 (0.84-0.98) | 0.014 | 0.92 (0.84-1.01) | 0.065 | 0.86 (0.79-0.93) | <0.001 |
| Cardiovascular mortality |  |  |  |  |  |  |
| Q1 (<1.33) | Reference | - | Reference | - | Reference | - |
| Q2 (1.34-1.37) | 0.74 (0.54-1.01) | 0.061 | 0.73 (0.53-0.99) | 0.045 | 0.68 (0.49-0.95) | 0.022 |
| Q3 (1.38-1.42) | 0.53 (0.37-0.74) | <0.001 | 0.50 (0.35-0.71) | <0.001 | 0.47 (0.32-0.67) | <0.001 |
| Q4 (>1.42) | 0.52 (0.36-0.73) | <0.001 | 0.53 (0.37-0.75) | <0.001 | 0.49 (0.33-0.71) | <0.001 |
| *P* for trend^*^ |  | <0.001 |  | 0.001 |  | <0.001 |
| Na/Cl ratio per 0.1 increase | 0.82 (0.75-0.90) | <0.001 | 0.81 (0.73-0.90) | <0.001 | 0.78 (0.70-0.87) | <0.001 |
| Model 1: unadjusted crude HR. Model 2: adjusted for age, sex, BMI, DM, and hypertension. Model 3: model 2 plus albumin, eGFR, cholesterol, calcium, potassium, HDL, LDL, Total Kt/V and centers. Q, quartile; BMI, body mass index; DM, diabetes mellitus; eGFR, estimated glomerular filtration rate; HDL, high-density cholesterol; LDL, low-density cholesterol; HR, hazards ratio; CI, confidence interval. ^*^, test for trend based on variable containing median value for each quartile. | | | | | | |

| Table S4. Association between Na/Cl ratio and cardiovascular mortality using competing risk models based on imputed data (n=3341). | | | | | | |
| --- | --- | --- | --- | --- | --- | --- |
|  | **Model 1** | ***P*-value** | **Model 2** | ***P*-value** | **Model 3** | ***P*-value** |
| Q1 (<1.33) | Reference | - | Reference | - | Reference | - |
| Q2 (1.34-1.37) | 0.75 (0.55-1.02) | 0.068 | 0.74 (0.54-1.01) | 0.06 | 0.71 (0.51-0.97) | 0.033 |
| Q3 (1.38-1.42) | 0.52 (0.37-0.74) | <0.001 | 0.50 (0.36-0.71) | <0.001 | 0.47 (0.33-0.68) | <0.001 |
| Q4 (>1.42) | 0.51 (0.36-0.72) | <0.001 | 0.51 (0.36-0.73) | <0.001 | 0.48 (0.33-0.69) | <0.001 |
| *P* for trend^*^ |  | <0.001 |  | <0.001 |  | <0.001 |
| Na/Cl ratio per 0.1 increase | 0.83 (0.76-0.91) | <0.001 | 0.81 (0.74-0.89) | <0.001 | 0.79 (0.72-0.88) | <0.001 |
| Model 1: unadjusted crude HR. Model 2: adjusted for age, sex, BMI, DM, and hypertension. Model 3: model 2 plus albumin, eGFR, cholesterol, calcium, potassium, HDL, LDL, Total Kt/V and centers. Q, quartile; BMI, body mass index; DM, diabetes mellitus; eGFR, estimated glomerular filtration rate; HDL, high-density cholesterol; LDL, low-density cholesterol; HR, hazards ratio; CI, confidence interval. ^*^, test for trend based on variable containing median value for each quartile. | | | | | | |

1. Analyses including left-censored participants and using parametric models for interval-censored survival-time data (stintreg).

| Table S5. Association between Na/Cl ratio and mortality using parametric models for interval-censored survival-time data based on complete data (n=2103). | | | | | | |
| --- | --- | --- | --- | --- | --- | --- |
|  | Model 1 | *P*-value | Model 2 | *P*-value | Model 3 | *P*-value |
| All-cause mortality |  |  |  |  |  |  |
| Q1 (<1.33) | Reference | - | Reference | - | Reference | - |
| Q2 (1.34-1.37) | 1.05 (0.83-1.35) | 0.671 | 1.13 (0.88-1.44) | 0.346 | 0.83 (0.64-1.08) | 0.171 |
| Q3 (1.38-1.42) | 1.25 (0.98-1.59) | 0.076 | 1.35 (1.05-1.72) | 0.017 | 0.92 (0.71-1.21) | 0.561 |
| Q4 (>1.42) | 0.89 (0.68-1.16) | 0.377 | 1.01 (0.78-1.32) | 0.918 | 0.65 (0.48-0.87) | 0.004 |
| *P* for trend^*^ |  | 0.827 |  | 0.412 |  | 0.012 |
| Na/Cl ratio per 0.1 increase | 0.98 (0.94-1.02) | 0.345 | 1.01 (0.92-1.10) | 0.857 | 0.79 (0.70-0.89) | <0.001 |
| Cardiovascular mortality |  |  |  |  |  |  |
| Q1 (<1.33) | Reference | - | Reference | - | Reference | - |
| Q2 (1.34-1.37) | 0.85 (0.58-1.23) | 0.387 | 0.87 (0.60-1.27) | 0.476 | 0.75 (0.50-1.12) | 0.158 |
| Q3 (1.38-1.42) | 0.88 (0.59-1.29) | 0.506 | 0.90 (0.61-1.33) | 0.606 | 0.76 (0.49-1.17) | 0.207 |
| Q4 (>1.42) | 0.49 (0.30-0.80) | 0.004 | 0.54 (0.33-0.88) | 0.013 | 0.43 (0.25-0.75) | 0.002 |
| *P* for trend^*^ |  | 0.009 |  | 0.028 |  | 0.004 |
| Na/Cl ratio per 0.1 increase | 0.86 (0.78-0.94) | 0.001 | 0.78 (0.67-0.92) | 0.002 | 0.68 (0.55-0.83) | <0.001 |
| Model 1: unadjusted crude HR. Model 2: adjusted for age, sex, BMI, DM, and hypertension. Model 3: model 2 plus albumin, eGFR, cholesterol, calcium, potassium, HDL, LDL and centers. Q, quartile; BMI, body mass index; DM, diabetes mellitus; eGFR, estimated glomerular filtration rate; HDL, high-density cholesterol; LDL, low-density cholesterol; HR, hazards ratio; CI, confidence interval. ^*^, test for trend based on variable containing median value for each quartile. | | | | | | |

| Table S6. Association between Na/Cl ratio and mortality using parametric models for interval-censored survival-time data based on imputed data (n=3429). | | | | | | |
| --- | --- | --- | --- | --- | --- | --- |
|  | Model 1 | *P*-value | Model 2 | *P*-value | Model 3 | *P*-value |
| All-cause mortality |  |  |  |  |  |  |
| Q1 (<1.33) | Reference | - | Reference | - | Reference | - |
| Q2 (1.34-1.37) | 0.84 (0.69-1.02) | 0.081 | 0.83 (0.68-1.01) | 0.065 | 0.70 (0.57-0.85) | <0.001 |
| Q3 (1.38-1.42) | 0.87 (0.72-1.06) | 0.166 | 0.86 (0.71-1.04) | 0.119 | 0.72 (0.59-0.88) | 0.001 |
| Q4 (>1.42) | 0.87 (0.72-1.05) | 0.156 | 0.9 (0.75-1.1) | 0.311 | 0.72 (0.59-0.89) | 0.002 |
| *P* for trend^*^ |  | 0.180 |  | 0.317 |  | 0.003 |
| Na/Cl ratio per 0.1 increase | 0.98 (0.94-1.02) | 0.345 | 0.96 (0.89-1.03) | 0.279 | 0.87 (0.81-0.94) | <0.001 |
| Cardiovascular mortality |  |  |  |  |  |  |
| Q1 (<1.33) | Reference | - | Reference | - | Reference | - |
| Q2 (1.34-1.37) | 0.77 (0.57-1.05) | 0.102 | 0.77 (0.57-1.05) | 0.103 | 0.71 (0.52-0.98) | 0.036 |
| Q3 (1.38-1.42) | 0.62 (0.45-0.86) | 0.004 | 0.61 (0.44-0.84) | 0.003 | 0.55 (0.39-0.78) | 0.001 |
| Q4 (>1.42) | 0.57 (0.41-0.80) | 0.001 | 0.58 (0.42-0.82) | 0.002 | 0.52 (0.36-0.75) | 0.001 |
| *P* for trend^*^ |  | <0.001 |  | <0.001 |  | <0.001 |
| Na/Cl ratio per 0.1 increase | 0.86 (0.78-0.94) | 0.001 | 0.84 (0.76-0.93) | 0.001 | 0.81 (0.73-0.89) | <0.001 |
| Model 1: unadjusted crude HR. Model 2: adjusted for age, sex, BMI, DM, and hypertension. Model 3: model 2 plus albumin, eGFR, cholesterol, calcium, potassium, HDL, LDL and centers. Q, quartile; BMI, body mass index; DM, diabetes mellitus; eGFR, estimated glomerular filtration rate; HDL, high-density cholesterol; LDL, low-density cholesterol; HR, hazards ratio; CI, confidence interval. ^*^, test for trend based on variable containing median value for each quartile. | | | | | | |

1. Sensitivity analyses

| Table S7. Association between Na/Cl ratio and mortality using sub-distribution hazard model based on imputed data. | | | | |
| --- | --- | --- | --- | --- |
| Outcomes | HR (95% CI) by Na/Cl ratio | | | |
|  | Q1 (<1.33) | Q2 (1.34-1.37) | Q3 (1.38-1.42) | Q4 (>1.42) |
| All-cause mortality |  |  |  |  |
| Patients without deaths during the first 2 years of follow-up | 1.0 | 0.68 (0.52-0.89) | 0.74 (0.57-0.96) | 0.70 (0.54-0.92) |
| Patients with follow-up period ≥ 24 months | 1.0 | 0.68 (0.52-0.89) | 0.74 (0.57-0.96) | 0.70 (0.54-0.92) |
| Cardiovascular mortality |  |  |  |  |
| Patients without deaths during the first 2 years of follow-up | 1.0 | 0.64 (0.42-0.96) | 0.51 (0.33-0.80) | 0.44 (0.27-0.71) |
| Patients with follow-up period ≥ 24 months | 1.0 | 0.63 (0.42-0.96) | 0.51 (0.33-0.80) | 0.44 (0.27-0.71) |
| Model adjusted for age, sex, BMI, DM, and hypertension. albumin, eGFR, cholesterol, calcium, potassium, HDL, LDL and centers. Q, quartile; BMI, body mass index; DM, diabetes mellitus; eGFR, estimated glomerular filtration rate; HDL, high-density cholesterol; LDL, low-density cholesterol; HR, hazards ratio; CI, confidence interval. | | | | |

| Table S8. Association between Na/Cl ratio and mortality using Cox proportional hazard models based on imputed data. | | | | |
| --- | --- | --- | --- | --- |
| Outcomes | HR (95% CI) by Na/Cl ratio | | | |
|  | Q1 (<1.33) | Q2 (1.34-1.37) | Q3 (1.38-1.42) | Q4 (>1.42) |
| All-cause mortality |  |  |  |  |
| Patients without deaths during the first 2 years of follow-up | 1.0 | 0.68 (0.52-0.88) | 0.73 (0.56-0.95) | 0.68 (0.52-0.89) |
| Patients with follow-up period ≥ 24 months | 1.0 | 0.68 (0.52-0.88) | 0.73 (0.56-0.95) | 0.68 (0.52-0.89) |
| Cardiovascular mortality |  |  |  |  |
| Patients without deaths during the first 2 years of follow-up | 1.0 | 0.64 (0.42-0.97) | 0.51 (0.33-0.8) | 0.43 (0.27-0.69) |
| Patients with follow-up period ≥ 24 months | 1.0 | 0.64 (0.42-0.97) | 0.51 (0.33-0.8) | 0.43 (0.27-0.69) |
| Model adjusted for age, sex, BMI, DM, and hypertension. albumin, eGFR, cholesterol, calcium, potassium, HDL, LDL and centers. Q, quartile; BMI, body mass index; DM, diabetes mellitus; eGFR, estimated glomerular filtration rate; HDL, high-density cholesterol; LDL, low-density cholesterol; HR, hazards ratio; CI, confidence interval. | | | | |
